# Discovery and validation of acetyl-L-carnitine in serum for diagnosis of major depressive disorder and remission status through metabolomic approach

**DOI:** 10.3389/fpsyt.2022.1002828

**Published:** 2022-11-15

**Authors:** Seungyeon Lee, Sora Mun, You-Rim Lee, Hyebin Choi, Eun-Jeong Joo, Hee-Gyoo Kang, Jiyeong Lee

**Affiliations:** ^1^Department of Senior Healthcare, Graduate School, Eulji University, Gyeonggi, South Korea; ^2^Department of Biomedical Laboratory Science, College of Health Sciences, Eulji University, Gyeonggi, South Korea; ^3^Department of Laboratory Medicine, Korea University Anam Hospital, Seoul, South Korea; ^4^Department of Neuropsychiatry, School of Medicine, Eulji University, Daejeon, South Korea; ^5^Department of Psychiatry, Uijeongbu Eulji Medical Center, Eulji University, Gyeonggi, South Korea

**Keywords:** metabolite, biomarker, major depressive disorder, acetylcarnitine, remission status, drug treatment

## Abstract

Major depressive disorder (MDD) is one of the most common psychiatric disorders that accompany psychophysiological and mood changes. However, the pathophysiology-based disease mechanism of MDD is not yet fully understood, and diagnosis is also conducted through interviews with clinicians and patients. Diagnosis and treatment of MDD are limited due to the absence of biomarkers underlying the pathophysiological mechanisms of MDD. Although various attempts have been made to discover metabolite biomarkers for the diagnosis and treatment response of MDD, problems with sample size and consistency of results have limited clinical application. In addition, it was reported that future biomarker studies must consider exposure to antidepressants, which is the main cause of heterogeneity in depression subgroups. Therefore, the purpose of this study is to discover and validate biomarkers for the diagnosis of depression in consideration of exposure to drug treatment including antidepressants that contribute to the heterogeneity of the MDD subgroup. In the biomarker discovery and validation set, the disease group consisted of a mixture of patients exposed and unexposed to drug treatment including antidepressants for the treatment of MDD. The serum metabolites that differed between the MDD patients and the control group were profiled using mass spectrometry. The validation set including the remission group was used to verify the effectiveness as a biomarker for the diagnosis of depression and determination of remission status. The presence of different metabolites between the two groups was confirmed through serum metabolite profiling between the MDD patient group and the control group. Finally, Acetylcarnitine was selected as a biomarker. In validation, acetylcarnitine was significantly decreased in MDD and was distinguished from remission status. This study confirmed that the discovered acetylcarnitine has potential as a biomarker for diagnosing depression and determining remission status, regardless of exposure to drug treatment including antidepressants.

## Introduction

Major depressive disorder (MDD) is one of the most common psychiatric disorders and is characterized by marked mood swings with sadness or irritability and psychophysiological changes such as sleep, appetite, and libido disturbances lasting for at least 2 weeks (1). MDD has a worldwide prevalence of 17% and is confirmed to be associated with metabolic changes such as cardiovascular disease and metabolic syndrome (2). The pathophysiology-based diagnosis and treatment of MDD are not fully understood and are associated with a lack of distinct biomarkers for MDD (3). To date, there is no test that can reliably diagnose MDD, and tests such as the Hamilton Rating Scale for Depression (HAMD) and Beck Depression Inventory (BDI) based on subjective interviews with clinicians and patients have been performed for reference (4). The lack of pathophysiology-based biomarkers for MDD limits its accurate diagnosis and treatment (5). Biomarkers based on biomolecular mechanisms that can be used for MDD diagnosis, prognosis, and treatment would be useful in providing an objective method for diagnosing MDD rather than a diagnostic method based on subjective evaluation.

Metabolomics is the study of small molecules (up to 1.5 kDa) called metabolites. Since metabolites are the final downstream products of transcription and translation, they are closest to the phenotype (6) and largely reflect environmental influences, nutritional requirements, effects of xenobiotics and drugs, stress, and various pathological or internal changes in biochemical pathways (7, 8). Owing to the importance of these metabolites in biological systems, metabolite analysis is increasingly used in various research fields because it can improve the understanding of many pathological processes through altered metabolic pathways (9, 10). Non-targeted metabolomics techniques enable unbiased analysis and differential comparison of all metabolites directly involved in biochemical activity in tissue or body fluid samples without prior knowledge of the metabolites and provide more information than target metabolomics (10). Targeted metabolomics techniques target and measure the metabolite of interest, and a high level of specificity and accuracy is considered (10). Metabolite analysis based on these techniques can be useful in the search for potential biomarkers because it can identify different biological systems in individuals and explore the evidence of heterogeneous clinical manifestations. High-performance liquid chromatography and mass spectrometry are the main analytical techniques used in metabolomics, enabling the profiling or quantification of metabolites in samples with high throughput, accuracy, and precision.

To date, to identify biomarkers for the diagnosis and treatment response of MDD, lipids (11–14), amino acids (15–18), amines (19) and neurotransmitters (20, 21) in samples such as plasma, serum, urine, and cerebrospinal fluid, as well as other biometabolites, have been studied. A recent meta-analysis of MDD and peripheral blood metabolite function (22) reported that the clinical application of most non-targeted or targeted metabolite studies investigating MDD was limited due to issues regarding sample size and consistency of results. In addition, a meta-analysis suggested that, in future MDD biomarker studies, it is important to consider the presence or absence of antidepressant exposure, which is a major cause of heterogeneity in metabolic changes in the depression subgroup. Therefore, in this study, patients diagnosed with MDD who were exposed to drug treatment, including antidepressants, were included in the disease group.

By profiling the serum metabolites of the patients with MDD and the control group, metabolites showing differences between the groups were identified as potential biomarkers; validation was then performed to verify the effectiveness of the potential serum metabolite markers for the diagnosis of depression and determination of remission status. The metabolite biomarkers discovered through this process could be used as biomarkers for the diagnosis of MDD and remission status, irrespective of exposure to drug treatment, including antidepressants.

## Experimental methods

### Chemicals and reagents

High-performance liquid chromatography-grade methanol, water, and acetonitrile were purchased from J.T. Baker (NJ, USA). Formic acid (mass spectrometry grade) was purchased from Fluka Analytical (Buchs, Switzerland). Four small molecules [sulfamethoxazole, ketoprofen, MES (4- morpholineethanesulfonic acid), glyphosate], and 4-(2-hydroxyethyl)-1- piperazineethanesulfonic acid (HEPES) were used for the spiking sample, and Acetyl-d3-L-carnitine hydrochloride used as the internal standard in selected reaction monitoring (SRM) analysis was purchased from Sigma-Aldrich (St. Louis, USA). Acetylcarnitine was purchased from Santa Cruz Biotechnology (TX, USA) and used for assay standard solution.

### Sample collection

**FIGURE 1:**
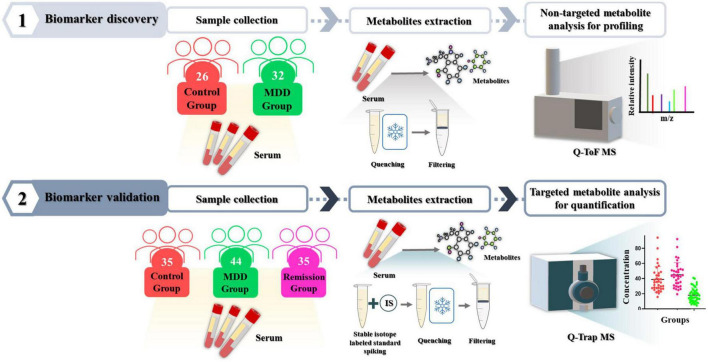
Experimental workflow. To discover metabolites that differed significantly between the depression patient group and the control group, serum metabolites were profiled using non-targeted metabolomic technique with Q-ToF mass spectrometry. Then, their effectiveness as biomarkers was validated in independent validation set using targeted metabolomic technique with Q-Trap mass spectrometry.

This study was approved by the Institutional Bioethics Committee of Eulji University (EMC 2016-08-009-010, October 10, 2016), and written informed consent was obtained from the participants. Depression severity was assessed using the Hamilton Depression Scale (17-item). The MDD patient group comprising the discovery and validation sets consisted of 76 drug-treated (DT) or non-drug treated (NDT) patients. The control group comprised 61 patients and the remission group consisted of 35 patients who achieved remission. The participants’ demographic data are presented in Table 1. All participants were 19 years of age or older. The drug-treated (DT) depression patient group included patients who received drug treatment including antidepressants for the treatment of depression, and the non-drug treated (NDT) group included patients who did not receive drug treatment. All participants in the control group consisted of healthy participants who did not smoke, had no head injury, had never taken psychiatric drugs, and no history of drug abuse, including alcohol, within 6 months. Blood (10 mL) was collected from the participants in a serum tube without anticoagulant and left for 30 min at room temperature to confirm coagulation, followed by centrifugation at 2,000 × g for 10 min. Serum, which was the supernatant, was separated and dispensed into a 1.5 mL micro tube and stored at –70°C for further analysis. Sample preparation for serum metabolite and untargeted/targeted metabolomic mass spectrometry methods performed in this study were based on previously established protocol (23, 24). The entire process involved in the analysis is illustrated in Figure 1.

**TABLE 1:** Demographics of participants.

Variables	Discovery set			Validation set			
		
	Control	MDD^a^	*P*-value	Control	MDD	Remission	*P*-value
Number of participants	26	32		35	44	35	
Age (mean ± SD^b^)	51 ± 8	52 ± 19	0.491^d^	65 ± 6	54 ± 15	62 ± 14	<0.05^e^
Sex (male/female)	6/20	12/20	0.238^f^	6/29	11/33	7/28	0.685^f^
Drug treated (DT)/non-drug treated (NDT)		14/18			26/18		
HAMD-17^c^ (mean ± SD^b^)		20 ± 5			21 ± 6		

^a^MDD, major depressive disorder; ^b^SD, standard deviation; ^c^HAMD-17, Hamilton Depression Scale (17-item); ^d^Mann–Whitney U-test; ^e^one-way ANOVA with Dunnet *t*-test for multiple comparison (MDD vs Control *p*-value and MDD vs Remission *p*-value were < 0.05, Control vs Remission *p*-value = 0.616); and ^f^Chi-square test.

#### Metabolite extraction and filtering

Thawed serum (100 μL) was dispensed into a 1.5 mL tube, and 200 μL of HPLC grade water was added and vortexed briefly. Then, 700 μL of cold 80% methanol was added, vortexed, and quenched by storage in a –70°C ultra-low temperature freezer. After quenching, the sample was vortexed for 1 min, sonicated for 10 min, left at room temperature for 10 min, and then centrifuged at 4°C for 10 min at 14,000 g. After centrifugation, 400 μL of the supernatant was added to a Nanosep filter. The Nanosep^®^ Centrifugal Device with Omega™ Membrane-3K (Pall Corporation, Port Washington, NY, USA) was activated with HPLC-grade water and 70% ethanol. The Nanosep filter tube containing the supernatant was centrifuged at 4°C for 20 min at 14,000 g, and the filtered sample solution was transferred to a new 1.5 mL tube. After the process of putting 400 μL of the sample solution into the Nanosep filter and obtaining the filtered sample solution, totally 800 μL of the filtered sample solution was completely dried in a vacuum concentrator (Scan Vac, LaboGene, Lynge, Denmark) to obtain a filtered sample solution and stored at –70°C until MS analysis.

#### Sample preparation for LC-MS/MS analysis

Five kinds of internal standards (MES, HEPES, and glyphosate concentrations of 20 μM, ketoprofen and sulfamethoxazole concentrations of 10 μM) and 80 μL of HPLC grade water mixed with 5% acetonitrile and 0.1% formic acid were added to the completely dried sample and resuspended. Subsequently, after 1 min of vortexing, 10 min of sonication, and 10 min of standing at room temperature, centrifugation was performed at 4°C for 10 min at 14,000 g. The obtained supernatant was diluted 10-fold with the resuspended solution and prepared in an MS vial. One microliter of each sample was used for the LC-MS/MS analysis. The mass spectrometry system was an Agilent 6546 quadrupole time-of-flight (Q-TOF) system (Agilent Technologies, Santa Clara, CA, USA) connected to an Agilent 1290 Infinity II liquid chromatography system. Agilent’s ZORBAX rapid resolution high definition stablebond SB-Aq (2.1 mm × 150 mm, 1.8 μm) column was used as the analysis column and Agilent ZORBAX StableBond-C8 (2.1 × 5 mm, 1.8 μm) column was used as the guard column. The columns were placed in a column oven maintained at 40°C and chromatographic separation was performed. The mobile phase A was water containing 0.1% formic acid and 5% acetonitrile whereas mobile phase B was acetonitrile containing 0.1% formic acid and 5% water. The flow rate of the mobile phase was 400 μL/min, and the gradient started with 100% A and decreased to 40% for 14 min, then further reduced to 35% for 3 min, 5% A was maintained for 5 min, and then for the last 5 min, a total of 30 min running method returning to 100% A was used. The Vcap voltage of the ion source capillary was 4,000 V, and the flow rate of drying nitrogen gas at a temperature of 225°C was 11 L/min. The scanning speed was 2 Hz, and data were obtained through MS scan (range < 1,700 m/z) and auto MS/MS scan (20–1,000 m/z) in positive ion mode.

#### Identification of metabolites that showed significant differences between major depressive disorder and control groups

Untargeted raw data obtained through mass spectrometry were imported into Profinder (version 10.0, Agilent Technologies), and molecular features (MF) were extracted. The CEF format file exported from Profinder was imported into the Mass Profiler Professional (version 15.1, Agilent Technologies) software, and alignment were performed. For alignment, the retention time window of 1% + 0.15 min and mass tolerance window of 20 ppm + 0 mDa were used. Subsequently, MFs from one condition and all samples were filtered based on frequency, and volcano plot were performed. In Mataboanaylst,^1^ partial least squares-discriminant analyses (PLS-DA) were conducted. Subsequently, targeted MS/MS analysis was performed, targeting the MFs listed in the volcano plot analysis and PLS-DA. Compound identification was performed using the METLIN database.^2^

### Sample preparation for biomarker candidate validation

The Participants constituting the validation set are independent of the discovery set. Thawed serum (100 μL) was dispensed into a 1.5 mL tube, and 100 μL of stable isotope-labeled standard solution 5μg/mL was added and vortexed briefly. After vortexing by adding 800 μL of cold 100% methanol, the sample tube was stored in a –70°C deep freezer for quenching. After quenching, the same procedure as the sample preparation process for LC-MS/MS analysis was performed. After filtering, the samples were completely dried in a vacuum concentrator then resuspended in 80 μL of 100% methanol. After 10 min of vortexing, 10 min of sonication, and 10 min of standing at room temperature, centrifugation was performed at 4°C for 10 min at 14,000 g. The supernatant obtained was diluted 100-fold with mobile phase A and prepared in an MS vial.

#### Validation of candidate biomarker through selected reaction monitoring

Selected reaction monitoring (SRM) was performed to validate candidate biomarker metabolites. After diluting the assay standard solution prepared in advance in mobile phase A, nine calibration solutions with concentrations of 5, 10, 50, 100, 150, 200, 250, and 500 ng/mL were prepared. To obtain parameter values, such as collision energy, as well as declustering, collision exit, and entrance potentials, compound optimization was performed, and SRM transitions were constructed. The SRM transition is shown in the form of precursor ion -> target ion, acetylcarnitine was 204.1- > 85.1 and Acetyl-d3-L-carnitine hydrochloride was 207.1- > 85.1. The SRM analysis was performed using SCIEX 5500 QTRAP and Exion LC (AB SCIEX, Foster City, CA, USA). An Agilent ZORBAX Eclipse Plus C18 (2.1 mm × 50 mm, 1.8 μm) was used as an analytical column, and an Agilent ZORBAX StableBond-C8 (2.1 mm × 5 mm, 1.8 μm) (Agilent, Santa Clara, CA, USA) was used as a guard column. Mobile phase A comprised water containing 0.1% formic acid and 5% acetonitrile and mobile phase B consisted of acetonitrile containing 0.1% formic acid and 5% water. The flow rate was set at 400 μL/min. The gradient of the mobile phase was initiated with 100% A, decreased to 0% at 5 min, and remained at 100% A from 7 to 10 min. The scan was performed in positive ion mode, and the machine parameters were as follows: ion-spray voltage, 5.5 kV; ion-source temperature, 500°C; nebulizer gas (Gas 1), nitrogen, 50 psi; turbo gas (Gas 2), nitrogen 50 psi; curtain gas, nitrogen 30 psi. The software used for data processing was Analyst Software version 1.6.1 (AB Sciex) and MultiQuant Software version 2.0.2 (AB Sciex). To evaluate the reproducibility of the SRM analysis, quality control samples having low, medium, and high concentrations of acetylcarnite in mobile phase A were prepared (Supplementary Table 1).

### Statistical analysis

IBM SPSS statistics version 26.0. (Armonk, NY: IBM Corp.) was used to confirm the statistical differences between the demographic information variables of the MDD group, the control group, and the remission group. The normality of the data was tested by the Shapiro–Wilk normality test. Then, based on the normality results, Mann-Whitney *U*-test and one-way ANOVA were used for continuous variables, and χ^2^ analyzes were used for categorical variables. Statistical analysis to detect differences in metabolites between the MDD patient group and the control group in the discovery set was performed using Mass Profiler Professional software. Through the filter by frequency function, MFs commonly detected in all samples in a group were filtered. The Shapiro–Wilk normality test was used to confirm the normality of the distribution of the filtered MFs. Based on the normality test result, the Mann-Whitney unpaired test was performed based on fold change > 2.0, and *p*-value < 0.05 for the MFs filtered by frequency. The multiple testing correction was set to Benjamini-Hochberg FDR for volcano plot analysis. Similarly, PLS-DA analysis was performed in Metaboanalyst (see text footnote 1) using the same MF list. The relative intensity and concentration quantitative values of metabolites obtained from mass spectrometry were analyzed statistically using GraphPad Prism software (version 8.4.2). Normality was tested using the Shapiro–Wilk normality test, and the unpaired *t*-test or Mann-Whitney test was conducted for quantitative comparison of metabolites between groups based on the normality test results.

## Results

### Metabolites showing a significant difference between the major depressive disorder and control groups

**FIGURE 2:**
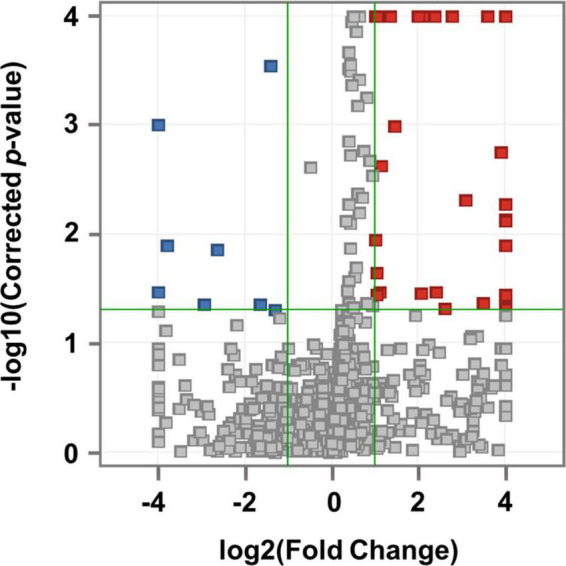
Volcano plot analysis. Compared to the control group, 53 MFs that increased in MDD are indicated by red squares and 8 MFs that decreased in MDD are indicated by blue squares. The horizontal green line represents the corrected *p*-value = 1.3 with the negative common logarithm applied, and the vertical green lines represent the standard value of fold change more than twice with the 2 logarithm (up-fold change = 1 and down-fold change = –1).

**FIGURE 3:**
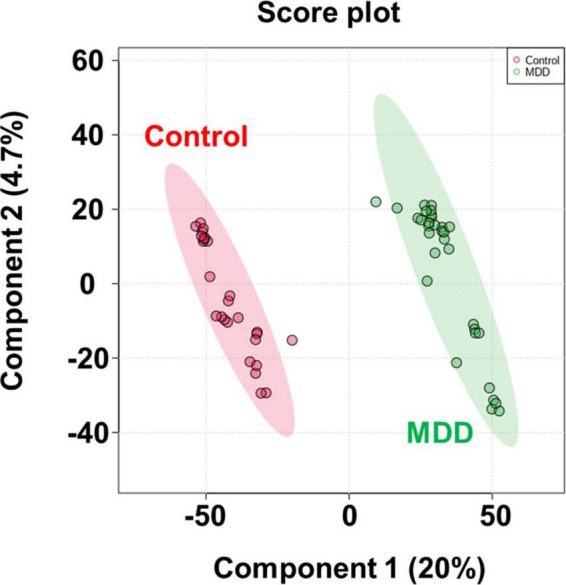
PLS-DA score plot. PLS-DA analysis to confirm group difference between control and MDD. The red dot indicates the control group, and the green dot indicates the MDD group.

**FIGURE 4:**
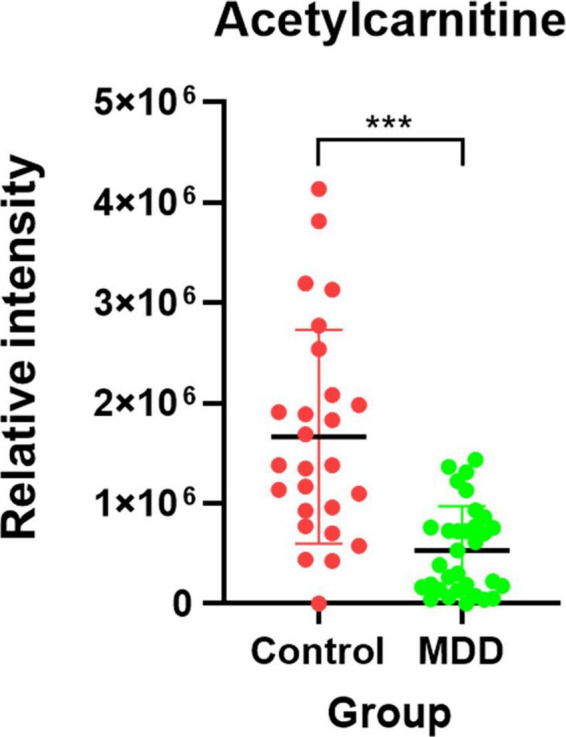
Candidate biomarker for diagnosis of MDD. The red dot indicates the control group, and the green dot indicates the MDD group. After the Shapiro-Wilk normality test, a difference test was performed through the Mann-Whitney test. Each red and green vertical line represents the standard deviation, and the central black line represents the mean. The schematic and statistical work were performed using GraphPad Prism software (version 8.4.2). ***P < 0.0001.

After importing the raw data obtained through the mass spectrometer into the MPP software and performing alignment, it was confirmed that 1,331 MFs were commonly detected in all samples under at least one condition after filtering by frequency. Parametric or non-parametric statistical techniques were used for comparisons between groups based on the Shapiro–Wilk normality test. The MF showing a significant difference between the MDD and control groups was confirmed through a volcano plot analysis of 1,331 MFs. There were 61 MFs satisfying a fold change > 2.0, and *p*-value < 0.05. Among them, 53 MFs showing an up-fold change and eight down-fold change MFs were identified (Figure 2). Subsequently, the filter by frequency list was equally used for PLS-DA analysis, and it was confirmed that the MDD and control groups were clearly distinguishable (Figure 3). MFs showing vip > 1 in PLS-DA and 61 MFs confirmed in volcano plot analysis were listed without duplicates for a total of 168 MFs. After obtaining the MS/MS scan data of the 168 MFs (Supplementary Table 2), compound identification was performed using the METLIN database. Among the 14 identified candidates, acetylcarnitine was finally selected by confirming whether it was endogenous in the blood, matched with the mass value, and exhibited a significant difference in intensity between the MDD and control groups (Figure 4).

### Validation of selected metabolite biomarkers through selected reaction monitoring

**FIGURE 5:**
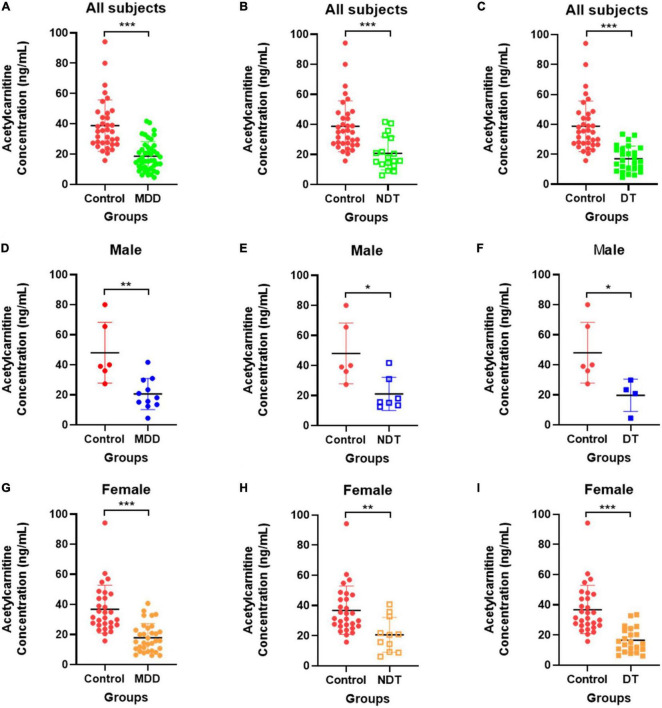
Validation of acetylcarnitine. **(A–C)** Shows the comparison between the overall control group and the whole MDD group, **(D–F)** shows the comparison within males, and **(G–I)** shows the comparison within females. Red indicates the control group, green indicates the MDD group, blue indicates male patients, and orange indicates female patients. Patients who did not receive drug treatment are indicated by unfilled squares, and those who received drug treatment are indicated by filled squares. After the Shapiro-Wilk normality test, the difference test was performed with the Mann-Whitney test or the unpaired *t*-test, and the GraphPad Prism software (version 8.4.2) was used for schematics and statistical work. The values in the graph represent mean ± SD. *P < 0.05, **P < 0.01, and ***P < 0.0001.

**FIGURE 6:**
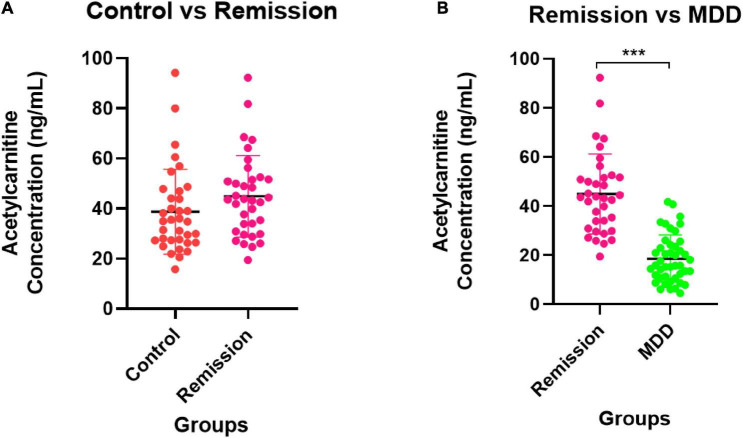
Validation for remission state of MDD patients. **(A)** The comparison of acetylcarnitine concentration between the control group and the remission group. **(B)** The comparison between the remission group and the MDD group. The control group is indicated by a red dot, the MDD group by a green dot, and the remission group by a pink dot. After the Shapiro–Wilk normality test, a difference test was performed through the Mann–Whitney test. The schematic and statistical work was performed using GraphPad Prism software (version 8.4.2). The values in the graph represent mean ± SD. ***P < 0.0001.

Figure 5 depicts the quantification results of acetylcarnitine selected as the final biomarker candidate in the validation set. A comparison of the entire control group with the entire MDD group (Figure 5A) showed that the MDD group had a decreased acetylcarnitine concentration compared to the control group, and a statistically significant difference was confirmed between the MDD and control groups. In addition, when all patients with MDD were divided into the NDT group, which is a group of patients without drug treatment, and the DT group, which received drug treatment, and the difference from the control group was compared (Figures 5B,C), acetylcarnitine was decreased in the MDD patient group compared to that in the control group. This was confirmed to be a statistically significant difference, regardless of exposure to drug treatment. Similarly, the results of screening and comparing all participants in the male control and male MDD groups (Figures 5D–F) and comparing all participants in the female control and female MDD groups (Figures 5G–I), compared to the control group, the MDD exhibited a lower concentration of acetylcarnitine and this was a statistically significant difference regardless of exposure to drug treatment. Compared to the acetylcarnitine concentration in the remission group, the MDD group showed a significantly lower concentration. This showed the same trend as in the comparison between the control and MDD groups (Figure 6A). In addition, the concentration of acetylcarnitine in the remission group was not significantly different from that in the control group (Figure 6B).

## Discussion

Acetylcarnitine, identified and verified as an MDD biomarker in this study, is naturally found in healthy humans in appropriate amounts. Acetylcarnitine is an ester of L-carnitine and acetate, and is synthesized by acetylcarnitine transferase in the human brain, liver, and kidney (25, 26). Acetylcarnitine promotes the uptake of acetyl-CoA into mitochondria during fatty acid oxidation, enhances acetylcholine production, stimulates protein and membrane phospholipid synthesis, and prevents excessive neuronal cell death by providing a substrate depot for cellular energy production (25). A number of studies have confirmed that acetylcarnitine is decreased in depression (3, 5, 27). These results confirm that acetylcarnitine is a potential diagnostic biomarker for depression. It has also been reported that endogenous l-carnitine-derived acetylacarnitine may serve as an antidepressant by improving brain energy metabolism and modulating neurotransmitters and neuroplasticity (5). Several previous studies demonstrated an association between MDD and acetylcarnitine. In a rodent model with characteristics similar to depression, decreased levels of acetylcarnitine and abnormal hippocampal glutamate function was confirmed (28). Depression and decreased levels of acetylcarnitine were also found in humans (3). In addition to the results of this study, a decrease in the level of endogenous acetylcarnitine confirmed in several studies can be an objective indicator of depression, and the antidepressant effect of acetylcarnitine supplementation has also been reported (5). In a recent study, acetylcarnitine was identified as a differential metabolite through the comparison of plasma metabolite profiling of the MDD and control groups using a metabolomic approach to identify MDD diagnostic biomarkers (27). However, validation using independent sample set was not conducted to evaluate the efficacy of the discovered biomarker candidates. In this study, considering antidepressant exposure, which is a major factor affecting the heterogeneity of the depression subgroup, the disease group comprised patients who were treated with drugs, including antidepressants, as well as drug-naive patients. Differential metabolites were identified by comparing the non-target metabolite profiles of MDD and control groups. Subsequently, it was validated through SRM to determine whether the discovered metabolite was effective as a biomarker for diagnosing MDD. Through the discovery process, similar to the results of previous studies, the tendency of the acetylcarnitine level to decrease significantly in the MDD group was confirmed. Similar to the discovery results, in the validation results, acetylcarnitine concentration was significantly lower in the MDD group than the control group and the remission group. In addition, there was no significant difference in acetylcarnitine levels between the remission and control groups. The sample size calculations were performed for this study considering the effect size 0.8 indicated in in a previous study (3), two-sided test, significance level of 0.05, and power of 0.8. Based on these parameters, at least 26 samples per group were required using Cohen’s d and power calculations (29). In this study, all MDD groups, control groups, and remission groups belonging to the discovery set and validation set were 26 or more each, so the minimum criterion to confirm the statistical significance of each group comparison was satisfied. However, the mean age differed significantly between the MDD group, the control group, and the remission group constituting the validation set (Table 1). In multiple comparisons of age variables, the differences between the MDD group and the control group and between the MDD group and the remission group were significant, but there was no difference between the control group and the remission group (Table 1). This indicates that there may be an effect on the age variable in the results of comparing the MDD group, each control group, and the remission group. If additional validation of acetylcarnitine is carried out in a follow-up study, taking into account the larger sample size, controlled subject variables, antidepressant type, and heterogeneity due to pharmacomechanism, the potential of acetylcarnitine as a biomarker for diagnosing depression and remission status will be further elucidated.

## Conclusion

In this study, serum acetylcarnitine was significantly lower in the MDD group, regardless of exposure to drug treatment, which differed significantly between the MDD group and the control and remission groups. We identified acetylcarnitine as a potential biomarker for diagnosing depression, determining remission status, and monitoring treatment effectiveness.

## Data availability statement

The raw data supporting the conclusions of this article will be made available by the authors, without undue reservation.

## Ethics statement

The studies involving human participants were reviewed and approved by the Institutional Bioethics Committee of Eulji University. The patients/participants provided their written informed consent to participate in this study.

## Author contributions

SL, SM, Y-RL, JL, and H-GK contributed to the study conception and design. SL, SM, Y-RL, HC, and E-JJ contributed to the conduct of the study and acquisition of data and analyzed and interpreted the data. SL drafted the manuscript. JL and H-GK supervised the research process and revised the manuscript for publication. All authors contributed to the manuscript and approved the submitted version.

## Footnotes

1
https://www.metaboanalyst.ca/

2
https://metlin-nl.scripps.edu/
