# Supplementary material for: Discovery and validation of acetyl-L-carnitine in serum for diagnosis of major depressive disorder and remission status through metabolomic approach
Source: Front Psychiatry. 2022 Nov 15;13:1002828. doi: 10.3389/fpsyt.2022.1002828 (PMC9707625; doi:10.3389/fpsyt.2022.1002828)
Supplement: Supplementary file 1 [file Table_1.docx]

**Supplementary table 1.** Reproducibility evaluation of SRM analysis using quality control sample

| **Acetylcarnitine concentration** | **Mean** | **SD^a^** | **CV(%)^b^** |
| --- | --- | --- | --- |
| QC^c^ Low (25 ng/mL) | 23.8 | 3.8 | 15.8 |
| QC^c^ Medium (75 ng/mL) | 70.4 | 8.2 | 11.7 |
| QC^c^ High (250 ng/mL) | 241.1 | 20.0 | 8.3 |

^a^SD, standard deviation; ^b^CV, coefficient of variation, ^c^QC, quality control
