# Supplementary material for: Discovery and validation of acetyl-L-carnitine in serum for diagnosis of major depressive disorder and remission status through metabolomic approach
Source: Front Psychiatry. 2022 Nov 15;13:1002828. doi: 10.3389/fpsyt.2022.1002828 (PMC9707625; doi:10.3389/fpsyt.2022.1002828)
Supplement: Supplementary file 2 [file Table_2.docx]

**Supplementary Table 2.** Integrated 168 MFs list from volcano plot analysis and PLS-DA

| **No.** | **Molecular feature  (mass@retantion time)** | **No.** | **Molecular feature  (mass@retantion time)** | **No.** | **Molecular feature  (mass@retantion time)** |
| --- | --- | --- | --- | --- | --- |
| 1 | 99.9745@0.53127474 | 29 | 657.4036@10.4154005 | 57 | 535.8841@17.968533 |
| 2 | 99.0683@1.8685569 | 30 | 651.4913@19.479155 | 58 | 502.3271@18.22174 |
| 3 | 984.8808@22.816572 | 31 | 649.8899@9.985617 | 59 | 495.3773@18.724695 |
| 4 | 961.6637@20.871103 | 32 | 649.3882@9.985991 | 60 | 495.3325@16.162043 |
| 5 | 955.2027@21.372215 | 33 | 637.3704@8.866903 | 61 | 475.7613@9.851189 |
| 6 | 899.6386@21.105417 | 34 | 636.7572@17.276539 | 62 | 469.413@20.5468 |
| 7 | 813.5077@18.118586 | 35 | 636.2982@19.910156 | 63 | 460.796@23.878933 |
| 8 | 788.8528@17.667496 | 36 | 630.4422@18.393555 | 64 | 458.3009@18.247787 |
| 9 | 761.5511@22.236362 | 37 | 622.7954@21.235527 | 65 | 456.2777@20.03239 |
| 10 | 752.3435@10.442589 | 38 | 620.2655@9.769904 | 66 | 455.3037@20.032042 |
| 11 | 751.5074@16.745167 | 39 | 618.9043@17.357988 | 67 | 451.3509@18.772327 |
| 12 | 746.3989@22.238787 | 40 | 613.4766@18.160511 | 68 | 448.2471@10.960406 |
| 13 | 729.5236@18.415472 | 41 | 605.1001@21.108278 | 69 | 445.252@7.2007294 |
| 14 | 725.0109@20.32075 | 42 | 597.1355@17.665838 | 70 | 444.7429@9.355107 |
| 15 | 721.1841@21.989523 | 43 | 596.8915@17.355244 | 71 | 443.3246@16.728989 |
| 16 | 715.4273@10.301888 | 44 | 595.3941@17.666676 | 72 | 443.2448@19.345058 |
| 17 | 710.1355@17.252867 | 45 | 593.6311@17.230154 | 73 | 442.2651@9.355693 |
| 18 | 695.892@10.197688 | 46 | 590.3008@16.808708 | 74 | 426.2325@19.348671 |
| 19 | 684.5023@18.106596 | 47 | 584.2809@8.0813265 | 75 | 420.2521@9.176109 |
| 20 | 682.4661@17.3354 | 48 | 577.1364@18.106775 | 76 | 418.2331@14.196352 |
| 21 | 681.3966@9.082589 | 49 | 574.4061@18.171974 | 77 | 407.78@24.017588 |
| 22 | 678.4319@18.129744 | 50 | 570.1283@17.237492 | 78 | 407.3249@18.828564 |
| 23 | 673.8789@10.093167 | 51 | 567.3686@18.113775 | 79 | 400.2591@17.521837 |
| 24 | 672.4405@17.613777 | 52 | 563.8134@9.490549 | 80 | 398.239@8.982616 |
| 25 | 671.9031@10.095624 | 53 | 561.3359@9.490618 | 81 | 397.3555@19.72797 |
| 26 | 670.4866@17.434973 | 54 | 559.3203@7.737513 | 82 | 395.4127@21.473558 |
| 27 | 665.5956@21.430521 | 55 | 556.3631@18.121075 | 83 | 394.213@19.345688 |
| 28 | 664.2918@10.017781 | 56 | 549.6042@17.249718 | 84 | 390.2019@13.526818 |
| **No.** | **Molecular feature  (mass@retantion time)** | **No.** | **Molecular feature  (mass@retantion time)** | **No.** | **Molecular feature  (mass@retantion time)** |
| 85 | 389.7256@8.9765415 | 113 | 203.1158@0.78648776 | 141 | 431.273@7.0728965 |
| 86 | 384.2642@18.838888 | 114 | 194.1154@18.275455 | 142 | 539.3227@9.34791 |
| 87 | 376.2259@8.775151 | 115 | 194.1154@1.671718 | 143 | 581.5859@22.882734 |
| 88 | 376.2221@19.48347 | 116 | 172.0712@1.1243708 | 144 | 299.1944@5.973142 |
| 89 | 369.2517@15.761416 | 117 | 169.0052@0.8268615 | 145 | 327.3139@19.180042 |
| 90 | 359.2079@8.821517 | 118 | 162.1045@18.039198 | 146 | 324.2202@10.509058 |
| 91 | 348.2875@16.945236 | 119 | 1550.2762@20.18763 | 147 | 737.4403@10.395099 |
| 92 | 330.1443@14.339512 | 120 | 153.9596@23.896944 | 148 | 1198.7653@21.866995 |
| 93 | 327.908@23.967537 | 121 | 143.9748@23.955492 | 149 | 248.1989@11.847571 |
| 94 | 313.2981@17.457476 | 122 | 1334.2802@19.973463 | 150 | 1278.2325@20.01313 |
| 95 | 299.0833@16.810406 | 123 | 1150.2076@19.822227 | 151 | 632.4477@18.731932 |
| 96 | 295.2511@17.123642 | 124 | 113.0841@2.613208 | 152 | 612.0787@17.773966 |
| 97 | 292.144@14.106929 | 125 | 112.9564@23.995382 | 153 | 436.3326@19.625175 |
| 98 | 288.2665@18.517576 | 126 | 108.9363@23.983452 | 154 | 641.4708@18.46342 |
| 99 | 284.2717@19.984726 | 127 | 143.0947@0.74013275 | 155 | 685.4976@18.437922 |
| 100 | 277.1782@7.2366204 | 128 | 287.9243@23.877283 | 156 | 628.4065@17.758152 |
| 101 | 276.1725@16.684042 | 129 | 198.9931@0.5257548 | 157 | 559.8729@17.955648 |
| 102 | 271.9522@23.873257 | 130 | 362.7824@23.999914 | 158 | 1014.2168@19.477776 |
| 103 | 271.2511@14.822287 | 131 | 603.3466@8.035851 | 159 | 349.298@19.270245 |
| 104 | 263.2069@18.581944 | 132 | 647.3728@8.304184 | 160 | 708.4426@17.435644 |
| 105 | 254.9368@23.873474 | 133 | 370.2203@6.5908504 | 161 | 421.283@19.524776 |
| 106 | 240.1727@11.734526 | 134 | 785.5931@19.586756 | 162 | 692.4682@17.63264 |
| 107 | 239.1886@14.038818 | 135 | 629.8529@9.869015 | 163 | 707.4821@16.74275 |
| 108 | 238.1416@2.7448664 | 136 | 691.3989@8.547655 | 164 | 1414.2653@20.041403 |
| 109 | 223.1784@8.134832 | 137 | 585.8265@9.620359 | 165 | 328.1463@17.242493 |
| 110 | 220.1463@17.43371 | 138 | 605.3621@9.749154 | 166 | 1257.304@19.998705 |
| 111 | 216.1726@14.051573 | 139 | 282.256@19.619253 | 167 | 1150.2072@19.768019 |
| 112 | 206.1519@8.135437 | 140 | 353.3295@19.889309 | 168 | 766.4847@18.0935 |
